# Utilizing fully-automated 3D organ segmentation for hepatic steatosis assessment with CT attenuation-based parameters

**DOI:** 10.1007/s00330-024-10660-4

**Published:** 2024-02-23

**Authors:** Jeongin Yoo, Ijin Joo, Sun Kyung Jeon, Junghoan Park, Soon Ho Yoon

**Affiliations:** 1 Department of Radiology, Seoul National University Hospital, 101 Daehak-ro, Jongno-gu, Seoul, 03080 Korea; 2 Department of Radiology, Seoul National University College of Medicine, 103 Daehak-ro, Jongno-gu, Seoul, 03080 Korea; 3 Institute of Radiation Medicine, Seoul National University Medical Research Center Seoul National University Hospital, 101 Daehak-ro, Jongno-gu, Seoul, 03080 Korea; 4 MEDICALIP. Co. Ltd., Seoul, Korea

**Keywords:** Fatty liver, Non-alcoholic fatty liver disease, Deep learning, Multidetector computed tomography

## Abstract

**Objectives:**

To investigate the clinical utility of fully-automated 3D organ segmentation in assessing hepatic steatosis on pre-contrast and post-contrast CT images using magnetic resonance spectroscopy (MRS)-proton density fat fraction (PDFF) as reference standard.

**Materials and methods:**

This retrospective study analyzed 362 adult potential living liver donors with abdominal CT scans and MRS-PDFF. Using a deep learning-based tool, mean volumetric CT attenuation of the liver and spleen were measured on pre-contrast (liver(L)_pre and spleen(S)_pre) and post-contrast (L_post and S_post) images. Agreements between volumetric and manual region-of-interest (ROI)-based measurements were assessed using the intraclass correlation coefficient (ICC) and Bland–Altman analysis. Diagnostic performances of volumetric parameters (L_pre, liver-minus-spleen (L-S)_pre, L_post, and L-S_post) were evaluated for detecting MRS-PDFF ≥ 5% and ≥ 10% using receiver operating characteristic (ROC) curve analysis and compared with those of ROI-based parameters.

**Results:**

Among the 362 subjects, 105 and 35 had hepatic steatosis with MRS-PDFF ≥ 5% and ≥ 10%, respectively. Volumetric and ROI-based measurements revealed ICCs of 0.974, 0.825, 0.992, and 0.962, with mean differences of −4.2 HU, −3.4 HU, −1.2 HU, and −7.7 HU for L_pre, S_pre, L_post, and S_post, respectively. Volumetric L_pre, L-S_pre, L_post, and L-S_post yielded areas under the ROC curve of 0.813, 0.813, 0.734, and 0.817 for MRS-PDFF ≥ 5%; and 0.901, 0.915, 0.818, and 0.868 for MRS-PDFF ≥ 10%, comparable with those of ROI-based parameters (0.735–0.818; and 0.816–0.895, Ps = 0.228–0.911).

**Conclusion:**

Automated 3D segmentation of the liver and spleen in CT scans can provide volumetric CT attenuation-based parameters to detect and grade hepatic steatosis, applicable to pre-contrast and post-contrast images.

**Clinical relevance statement:**

Volumetric CT attenuation-based parameters of the liver and spleen, obtained through automated segmentation tools from pre-contrast or post-contrast CT scans, can efficiently detect and grade hepatic steatosis, making them applicable for large population data collection.

**Key Points:**

• *Automated organ segmentation enables the extraction of CT attenuation-based parameters for the target organ.*

• *Volumetric liver and spleen CT attenuation-based parameters are highly accurate in hepatic steatosis assessment.*

• *Automated CT measurements from pre- or post-contrast imaging show promise for hepatic steatosis screening in large cohorts.*

**Graphical abstract:**

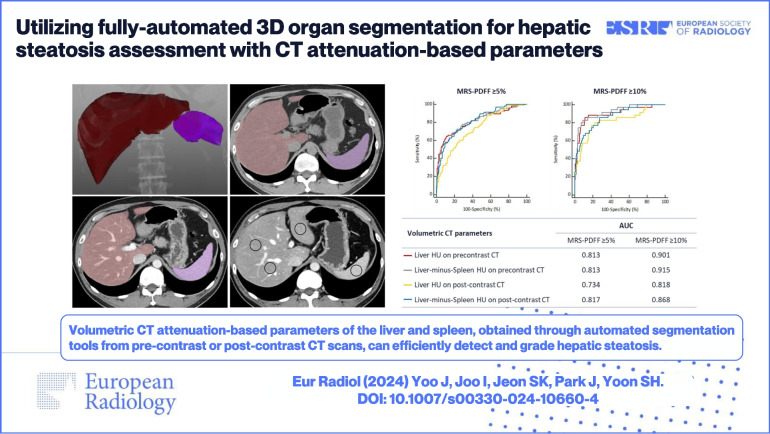

**Supplementary Information:**

The online version contains supplementary material available at 10.1007/s00330-024-10660-4.

## Introduction

Hepatic steatosis, characterized by fat accumulation in the hepatocytes, is a key feature of non-alcoholic fatty liver disease. Liver biopsy is traditionally the gold standard for detecting and grading hepatic steatosis [1]. However, its invasive nature makes it less practical for routine and repeated use. In recent practice, non-invasive imaging techniques are increasingly preferred in clinical practice as valuable alternatives to invasive biopsy for evaluating and monitoring hepatic steatosis. Magnetic resonance spectroscopy (MRS) or chemical shift-encoded magnetic resonance imaging (MRI) for proton density fat fraction (PDFF) have emerged as non-invasive reference standards for hepatic steatosis, providing highly accurate and reliable measurements [2, 3]. A prior validation study with ex vivo human liver demonstrated that MRI-PDFF exhibited excellent correlation with MRS-PDFF (*r *= 0.984) and strong correlations with histological steatosis grades (*r *= 0.850) and extracted triglycerides (*r *= 0.871) [4]. Moreover, quantitative ultrasound techniques have shown promise for quantitatively assessing hepatic steatosis [5]. In contrast, computed tomography (CT) is not the method of choice for hepatic steatosis evaluation owing to its reliance on ionizing radiation and its limited performance in assessing mild hepatic steatosis. Nevertheless, CT is extensively used in various clinical investigations and is a cost-effective opportunistic screening tool for hepatic steatosis [6]. CT-based hepatic steatosis assessment relies on the different X-ray absorptions between triglycerides and normal liver tissue, resulting in decreased CT attenuation values as hepatic steatosis severity increases [7]. Parameters for hepatic steatosis assessment involve the absolute CT attenuation value of the liver [8] and the CT attenuation difference between the liver and spleen [9]. Their area under the receiver operating characteristic (ROC) curve (AUC) was reported to be approximately 0.7 for detecting pathological steatosis > 5% and approximately 0.9 for > 33%, respectively [10]. These measurements are often obtained using a region-of-interest (ROI) approach, with a preference for pre-contrast CT [2]. However, post-contrast CT can also be utilized [11].

Recently, major advancements in deep learning algorithms have led to the emergence of automated medical image segmentation [12]. These techniques are widely employed to efficiently and objectively obtain body composition data, organ volumes, and radiomics features [13]. Furthermore, these techniques have been applied for hepatic steatosis assessment, as demonstrated in a recent study that utilized organ segmentation-based volumetric CT attenuation measurements in a large screening cohort [14]. Volumetric CT attenuation measurements may provide a more comprehensive representation of the overall characteristics of the liver, even in cases with uneven steatosis. However, despite the many previous studies reporting the accuracy of ROI-based CT attenuation measurements for evaluating hepatic steatosis [10, 11, 15, 16], data regarding the accuracy of volumetric CT attenuation measurements using robust reference standards and their potential interchangeability with ROI-based measurements are limited.

Therefore, this study aimed to investigate the clinical utility of fully automated three-dimensional (3D) organ segmentation in assessing hepatic steatosis on pre-contrast and post-contrast CT images using MRS-PDFF as the reference standard.

## Methods

### Ethics statement

Our institutional review board approved this retrospective study and waived the requirement for informed consent because of the retrospective nature of the study.

### Study population

We searched the radiologic database in our institution to identify adults who underwent liver CT and MRI between January 2017 and June 2021 as part of a preoperative work-up for potential living liver donors. We excluded the following subjects: (1) those under 18 years old and (2) those with a time interval of more than 60 days between the CT and MRI scans. None of the subjects had a history of liver or spleen surgery. During the study period, preoperative liver CT scans for potential living liver donors were routinely performed in our institution, using a dual-source, dual-energy (80 kVp and 150 kVp) CT scanner (Somatom Force, Siemens Healthineers, Erlangen, Germany). These scans included pre-contrast, arterial, portal venous, and delayed phase images. Additionally, preoperative liver MRI scans were performed using a 3.0-T magnetic resonance scanner (MAGNETOM Skyra, Siemens Healthineers) equipped with a 60-channel torso phased-array coil. These MRI scans encompassed the measurement of MRS-derived PDFF, a non-invasive gold standard for quantifying liver fat [2]. Fat fraction spectroscopy measurements were conducted with a modified stimulated-echo acquisition sequence at various echo times within the single voxel in the dome area of segment VII or VIII of the liver. The voxel was carefully positioned to avoid large blood vessels, bile ducts, and liver edges. Supplementary 1 describes detailed scan protocols of the liver CT and MRS-PDFF.

### CT attenuation values of the liver and spleen

**Fig. 1:**
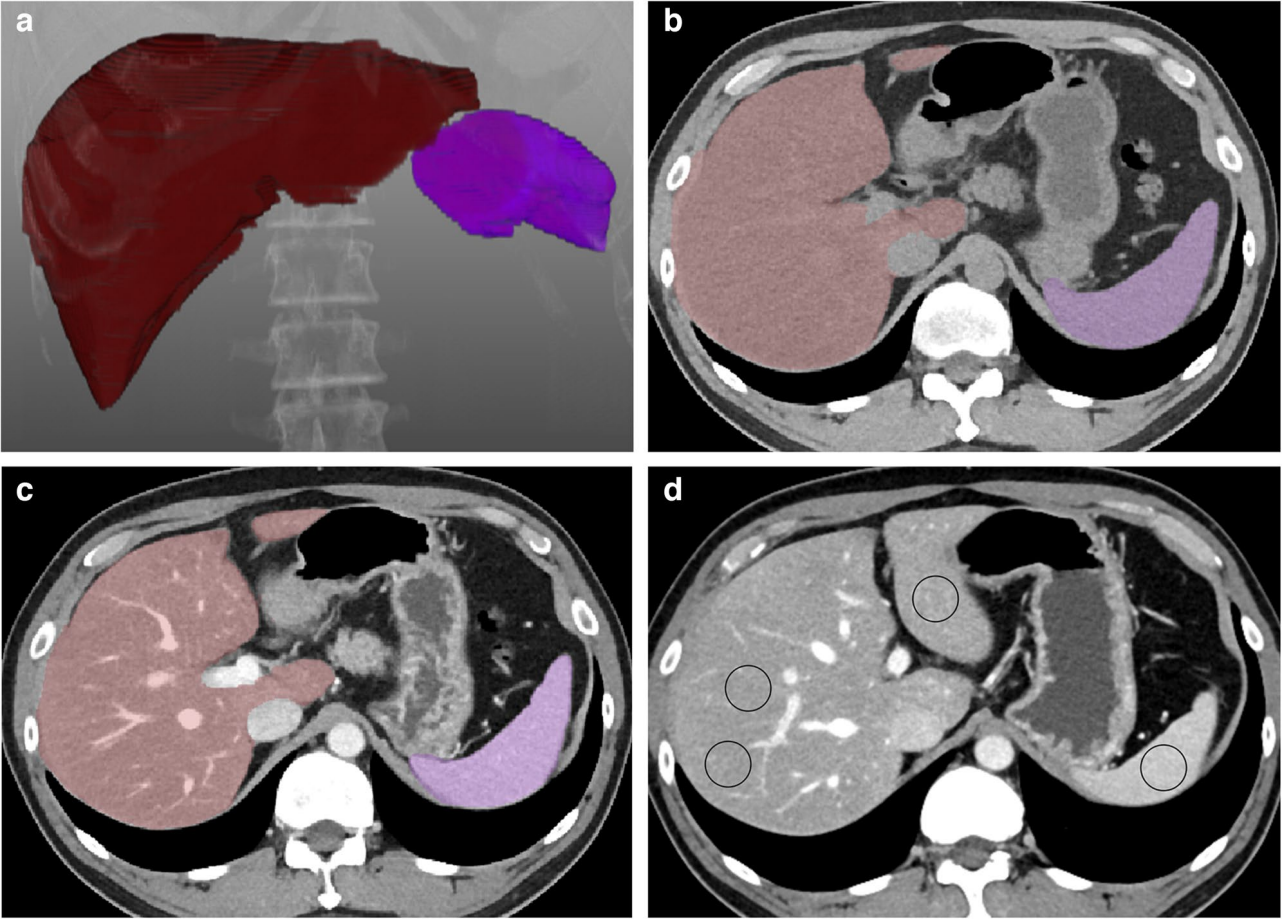
An illustration of volumetric and ROI-based measurement of liver HU and spleen HU in a 50-year-old man whose MRS-PDFF of the liver was 25.7%. The fully automated segmentation of the liver and spleen was conducted on pre-contrast and post-contrast abdominal CT scan (**a**: 3D volume rendering image, **b**: axial pre-contrast CT image, **c**: axial post-contrast CT image). The calculated volumetric mean CT attenuation values of the liver and spleen were 36 HU and 55 HU on pre-contrast CT images and 105 HU and 147 HU on post-contrast CT images, respectively. **d** ROI-based measurements of the liver HU and spleen HU were performed using three ROIs in the liver and one ROI in the spleen, respectively. *MRS-PDFF = magnetic resonance spectroscopy-proton density fat fraction; ROI = region-of-interest*

Two methods were utilized to assess the CT attenuation values in Hounsfield units (HU) of the liver and spleen: a two-dimensional manual ROI-based method and a 3D automated volumetric method (Fig. 1). Measurements were conducted on pre-contrast and post-contrast images acquired during the portal venous phase, which were reconstructed using a linear blending with a 6:4 ratio of 80 kVp and tin filtered 150 kVp. Using each method, the mean HU values of the liver and spleen were determined from pre-contrast images (L_pre and S_pre, respectively) and post-contrast images (L_post and S_post, respectively). Detailed procedures for each method are described below.

#### Manual ROI-based measurement

A board-certified radiologist (J.Y.) with 9 years of experience in body imaging manually positioned three circular ROIs in the liver, specifically in the left lobe, right anterior section, and right posterior section of each subject, following the methodology from a prior study [15]. Additionally, a circular ROI was placed in the central portion of the splenic parenchyma. Precautions were taken to ensure the ROIs had sizes of 250–300 mm^2^ and were positioned in regions free from hepatic vessels, focal lesions, and other causes of heterogeneity, including artifacts. The picture archiving and communication system (INFINITT PACS M6, INFINITT Healthcare) was utilized to draw the ROIs. The ROIs were initially drawn on the post-contrast images (Fig. 1). Subsequently, they were transferred to the corresponding pre-contrast images with minor adjustments when required, ensuring consistent anatomical positioning across both sets of images. The average HU values of the three liver ROIs were separately calculated for the pre- and post-contrast images, denoted as L_pre and L_post, respectively. Similarly, the mean HU values of the spleen ROI were denoted as S_pre and S_post, respectively.

#### Volumetric measurement using a 3D multi-organ segmentation algorithm

A commercially available multi-organ segmentation program (MEDIP PRO v2.4.0.0, MEDICAL IP Co. Ltd.), developed based on the 3D nnU-Net algorithm, was utilized to obtain volumetric CT attenuation measurements for the liver and spleen. This program was used to volumetrically segment the liver and spleen, generating a 3D mask that represented these organs while not excluding vascular structures within the organ. Subsequently, the volumetric mean of CT attenuation was automatically computed for all designated liver and spleen voxels from pre-contrast and post-contrast images, respectively.

### CT attenuation-based parameters for assessing hepatic steatosis

Based on the previous CT studies on hepatic steatosis assessment [10, 11, 14], we examined four CT attenuation-based parameters. These parameters comprised liver HU values (L_pre and L_post) and parameters quantifying the difference between liver and spleen HU (Liver-minus-Spleen [L-S]_pre and L-S_post).

### Statistical analysis

All statistical analyses were performed using IBM SPSS Statistics for Windows (version 25.0, IBM Corp) and MedCalc Statistical Software (version 18.9.1, MedCalc Software bvba). *p* values less than 0.05 were considered statistically significant.

The agreements between volumetric and manual ROI-based measurements of CT attenuation values of the liver and spleen were assessed using the intraclass correlation coefficient (ICC) and Bland–Altman analysis. Based on the ICC estimate, values ˂ 0.5, 0.5–0.75, 0.75–0.9, and ˃ 0.90 indicated poor, moderate, good, and excellent agreements, respectively [17]. The correlations between volumetric CT attenuation-based parameters for hepatic steatosis (L_pre, L-S_pre, L_post, and L-S_post) and MRS-PDFF values were investigated using Pearson’s correlation analysis. The diagnostic performances of these parameters were evaluated using ROC curve analysis to identify MRS-PDFF values ≥ 5% (indicating mild steatosis) and ≥ 10% (indicating moderate to severe steatosis) [18]. The AUC was compared between parameters obtained from the same imaging phase (L_pre vs. L-S_pre and L_post vs. L-S_post) and between the same type of parameters obtained from different imaging phases (L_pre vs. L_post; L-S_pre vs. L-S_post) using z-statistics. Additionally, these parameters were compared with the ROI-based parameters using z-statistics. The cutoff values for volumetric CT attenuation-based parameters were determined by maximizing the Youden index, and the corresponding sensitivity and specificity were calculated.

## Results

### Study population

The final study population comprised 362 adults, including 208 men and 154 women, with a mean age of 37.3 years (Table 1). The subjects had a mean MRS-PDFF value of 4.8% (range, 0.4%–35.7%). Based on the MRS-PDFF values, 257 (71.0%) subjects did not have hepatic steatosis (MRS-PDFF < 5%), whereas 105 (29.0%) had hepatic steatosis (MRS-PDFF ≥ 5%), including 35 with moderate to severe hepatic steatosis (MRS-PDFF ≥ 10%).

**Table 1:** Characteristics of the study population

Characteristics	Values
Age (y), mean ± SD (range)	37.3 ± 11.5 (18–65)
Sex, no. (%) of individuals	
Men	208 (57.5%)
Women	154 (42.5%)
MRS-PDFF (%)	
Mean ± SD (range)	4.8 ± 4.5 (0.4–35.7)
No. (%) of individuals	
< 5%	257 (71.0%)
≥ 5% and < 10%	70 (19.3%)
≥ 10%	35 (9.7%)

*SD*, standard deviation; *MRS*, magnetic resonance spectroscopy; *PDFF*, proton density fat fraction

### Agreements between volumetric and ROI-based measurements of CT attenuation values

The volumetric measurements of L_pre and L_post demonstrated excellent agreements with the ROI-based measurements, with ICC values of 0.974 (95% confidence interval [CI]: 0.968–0.979) and 0.992 (95% CI: 0.990–0.994), respectively and mean differences of −4.2 HU and −1.2 HU, respectively (Supplementary Fig. 1).

The volumetric measurements of S_pre showed good agreement with the ROI-based measurements, with an ICC of 0.825 (95% CI: 0.785–0.858), and S_post exhibited excellent agreement, with an ICC of 0.962 (95% CI: 0.953–0.969). The mean differences between the two methods for S_pre and S_post were −3.4 HU and −7.7 HU, respectively (Supplementary Fig. 1).

### Correlation of volumetric CT attenuation-based parameters and MRS-PDFF values

**Fig. 2:**
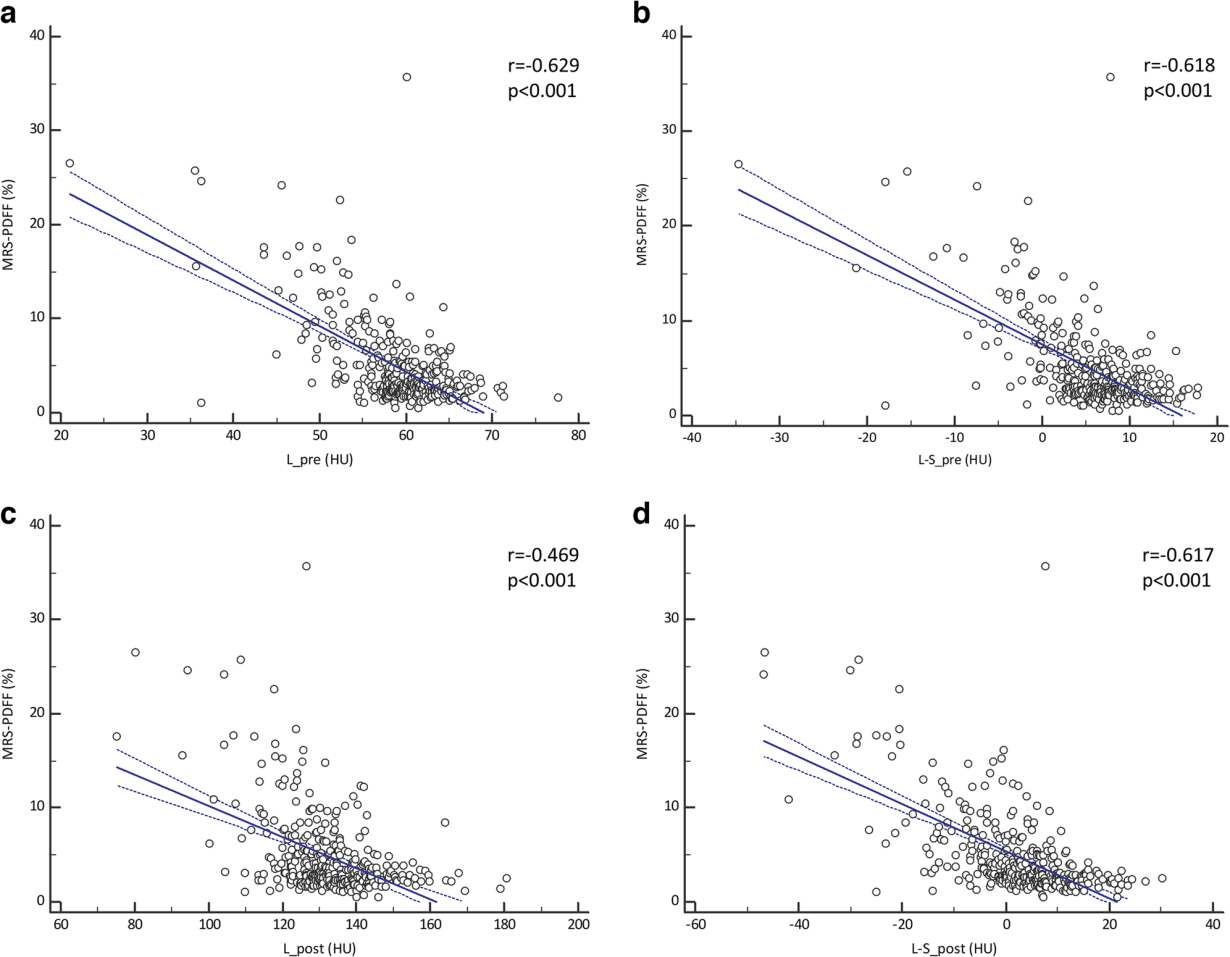
Scatter plots showing the fitted regression line (solid line) and 95% confidence interval (dotted lines) of volumetric CT attenuation-based parameters (**a**: L_pre, **b**: L-S_pre, **c**: L_post, **d**: L-S_post) with MRS-PDFF values. “r” represents Pearson’s correlation coefficient. *MRS-PDFF = magnetic resonance spectroscopy-proton density fat fraction; L_pre and L_post = mean liver HU on pre- and post-contrast CT images, respectively; L-S_pre and L-S_post = difference in mean HU between the liver and the spleen on pre- and post-contrast CT images, respectively*

All assessed volumetric CT attenuation-based parameters for hepatic steatosis (L_pre, L-S_pre, L_post, and L-S_post) showed significant negative correlations with MRS-PDFF values (*p *< 0.001). Their correlation coefficients with MRS-PDFF were −0.629 (95% CI: −0.681 to −0.554), −0.618 (95% CI: −0.678 to −0.550), −0.469 (95% CI: −0.546 to −0.384), and −0.617 (95% CI: −0.677 to −0.548) for L_pre, L-S_pre, L_post, and L-S_post, respectively (Fig. 2).

### Performances of CT attenuation-based parameters for diagnosing hepatic steatosis

**Fig. 3:**
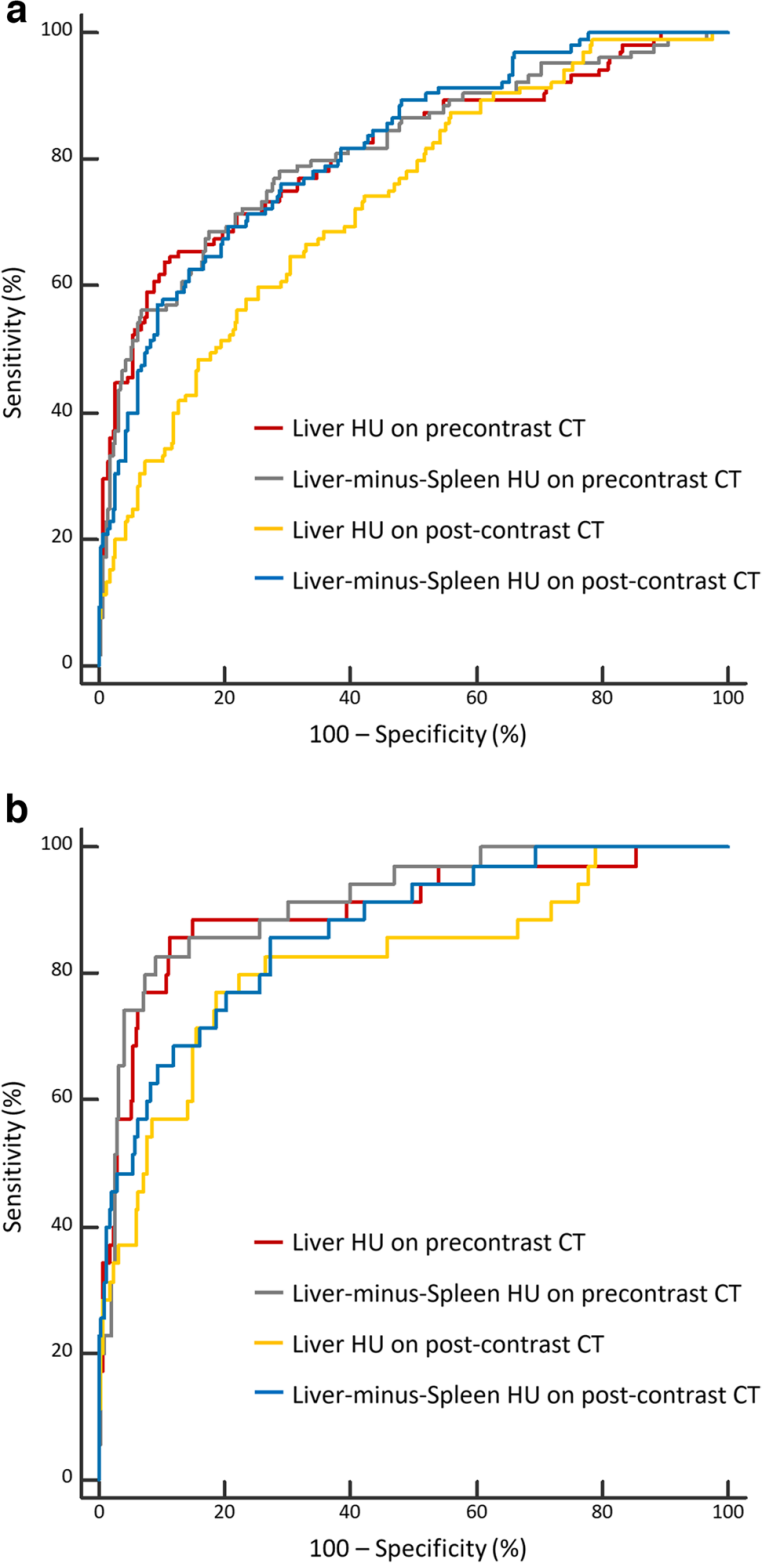
Graphs showing the area under the receiver operating characteristic curve of volumetric CT attenuation-based parameters to identify MRS-PDFF ≥ 5% (**a**) and ≥ 10% (**b**). *MRS-PDFF = magnetic resonance spectroscopy-proton density fat fraction*

Volumetric L_pre, L-S_pre, L_post, and L-S_post, demonstrated AUCs of 0.813, 0.813, 0.734, and 0.817, respectively, for MRS-PDFF ≥ 5%; and 0.901, 0.915, 0.818, and 0.868, respectively, for MRS-PDFF ≥ 10% (Table 2) (Fig. 3). L_post exhibited the lowest AUC among the parameters for MRS-PDFF ≥ 5% and ≥ 10%, which was significantly lower than those of L_pre (*p* = 0.015) and L-S_post (*p* = 0.001) for MRS-PDFF ≥ 5% (Supplementary Table 1). However, the difference in their AUCs was insignificant for MRS-PDFF ≥ 10%.

**Table 2:** Diagnostic performances of CT attenuation-based parameters for hepatic steatosis

Hepatic steatosis	CT parameters	AUC (95% confidence interval)		*p* value
		Volumetric measurements	ROI-based measurements	
MRS-PDFF ≥ 5%	L_pre	0.813 (0.769–0.852)	0.803 (0.759–0.843)	0.395
	L-S_pre	0.813 (0.769–0.852)	0.818 (0.774–0.856)	0.761
	L_post	0.734 (0.685–0.778)	0.735 (0.686–0.779)	0.885
	L-S_post	0.817 (0.773–0.855)	0.813 (0.769–0.852)	0.718
MRS-PDFF ≥ 10%	L_pre	0.901 (0.866–0.930)	0.882 (0.844–0.913)	0.254
	L-S_pre	0.915 (0.881–0.942)	0.895 (0.859–0.925)	0.228
	L_post	0.818 (0.774–0.856)	0.816 (0.772–0.855)	0.911
	L-S_post	0.868 (0.829–0.901)	0.856 (0.816–0.901)	0.383

*AUC*, area under the receiver operating characteristic curve; *MRS-PDFF*, magnetic resonance spectroscopy-proton density fat fraction; *L_pre and L_post*, mean liver HU on pre- and post-contrast CT images, respectively; *L-S_pre and L-S_post*, difference in mean HU between the liver and the spleen on pre- and post-contrast CT images, respectively

Compared with the ROI-based parameters, all assessed volumetric CT attenuation-based parameters showed no significant differences (*p* = 0.228 to 0.911) (Table 2). The AUCs of ROI-based parameters were 0.803, 0.818, 0.735, and 0.813 for L_pre, L-S_pre, L_post, and L-S_post, respectively, for MRS-PDFF ≥ 5% and 0.882, 0.895, 0.816, and 0.856, respectively, for MRS-PDFF ≥ 10%.

Table 3 presents the cutoff values for each volumetric parameter, determined using the Youden index, for predicting MRS-PDFF ≥ 5% and ≥ 10%. Proposed cutoff values of L_pre and L-S_pre were 55.4 HU and 1.2 HU, respectively, for diagnosing MRS-PDFF ≥ 10%. These cutoff values corresponded to a sensitivity and specificity of 85.7% and 88.7%, respectively, for L_pre and 82.9% and 90.8%, respectively, for L-S_pre.

**Table 3:** Application of proposed cutoffs of volumetric CT attenuation-based parameters

CT parameters	Cutoff (HU)	Sensitivity (%)	Specificity (%)
MRS-PDFF ≥ 5%			
L_pre	57.4	64.8 (68/105)	88.7 (228/257)
L-S_pre	4.2	68.6 (72/105)	82.5 (212/257)
L_post	127.9	58.1 (61/105)	76.7 (197/257)
L-S_post	0	69.5 (73/105)	79.4 (204/257)
MRS-PDFF ≥ 10%			
L_pre	55.4	85.7 (30/35)	88.7 (290/327)
L-S_pre	1.2	82.9 (29/35)	90.8 (297/327)
L_post	125.6	77.1 (27/35)	81.3 (266/327)
L-S_post	-0.3	85.7 (30/35)	72.5 (237/327)

*MRS-PDFF*, magnetic resonance spectroscopy-proton density fat fraction; *L_pre and L_post*, mean liver HU on pre- and post-contrast CT images, respectively; *L-S_pre and L-S_post*, difference in mean HU between the liver and the spleen on pre- and post-contrast CT images, respectively. Numbers in parentheses indicate the number of subjects used to calculate the percentage

## Discussion

Our study demonstrated that volumetric CT attenuation measurements, specifically the mean HU of the liver and the difference in mean HU between the liver and spleen, obtained using a fully automated segmentation tool, exhibited good diagnostic performance for detecting and grading hepatic steatosis in pre-contrast and post-contrast CT scans. The volumetric parameters, L_pre, L-S_pre, L_post, and L-S_post, correlated positively with MRS-PDFF values and yielded good AUC values (0.813, 0.813, 0.734, and 0.817, respectively) for detecting hepatic steatosis, defined as MRS-PDFF ≥ 5%. Additionally, these parameters achieved higher AUCs (0.901, 0.915, 0.818, and 0.868, respectively) for identifying moderate to severe hepatic steatosis, defined as MRS-PDFF ≥ 10%. The performances of the volumetric parameters were comparable with those of the ROI-based parameters, exhibiting AUCs ranging from 0.735 to 0.818 for MRS-PDFF ≥ 5% and 0.816 to 0.895 for MRS-PDFF ≥ 10%. This result suggests that automated volumetric measurements can be a viable alternative to the labor-intensive and time-consuming manual ROI-based approach in hepatic steatosis assessment. Given the increasing interest in using CT attenuation-based parameters for screening, our study findings support the applicability of the fully automated segmentation tool.

Previous studies on hepatic steatosis assessments using CT images have extensively investigated the manually ROI-measured HU of the liver [8, 10, 11, 19]. Additionally, the spleen has commonly served as an internal reference for normalization, with the difference between the liver and spleen HU or their ratio being explored as indicators of hepatic steatosis [8, 10, 20]. This explains why we selected the mean HU of the liver and the difference in mean HU between the liver and spleen as the volumetric parameters for this study. Previous ROI-based studies have demonstrated favorable diagnostic performance of these CT attenuation-based parameters for hepatic steatosis, using pathological findings [8, 10, 11, 19] or PDFF [15, 21, 22] as reference standards. However, their performance varies based on the degree of steatosis being targeted [11, 15, 20] or the specific imaging phase employed [11]. In our study, volumetric CT attenuation-based parameters exhibited slightly higher performances for MRS-PDFF ≥ 10% than for MRS-PDFF ≥ 5%. This finding is consistent with previous ROI-based studies [11, 15] and the well-acknowledged understanding that CT has limited sensitivity in detecting mild hepatic steatosis [23]. Our study also revealed that the non-normalized parameter (L_post) demonstrated significantly inferior performance in identifying MRS-PDFF ≥ 5% with post-contrast CT than the normalized parameter (L-S_post). Conversely, pre-contrast parameters, L_pre and L-S_pre, demonstrated comparable performances for MRS-PDFF ≥ 5% and ≥ 10%, suggesting that normalization is less important in pre-contrast CT. Nevertheless, as our study exclusively utilized a identical kVp and scanner setting, CT attenuation values might be influenced by factors such as kVp and scanner variations [24, 25]. Therefore, normalization may be necessary, even for pre-contrast CT scans, when collecting data from scan settings different from our study’s configuration. Our study findings revealed that L-S_post exhibited performances comparable with pre-contrast parameters, implying that post-contrast CT images can be utilized for hepatic steatosis assessment, consistent with a previous ROI-based study [11]. However, this result should be cautiously interpreted because liver and spleen attenuation measurements on post-contrast CT can be influenced by slight variations in scan timing, even within the same portal venous phase [8, 11], which poses challenges in attaining reproducible measurements.

Utilizing parameters derived from automated organ segmentation through deep learning techniques for liver steatosis evaluation is currently an actively researched topic [14, 26–28]. This approach is supported by data demonstrating a strong correlation between volumetric CT attenuation measurements and the extensively validated ROI-based measurement methods [14]. However, while direct research on the diagnostic accuracy of volumetric CT attenuation is limited, our study holds significance in providing foundational evidence. A recent study [14] showed excellent agreement between volumetric and ROI-based measurement methods for liver HU in pre-contrast CT scans, with a mean difference of 2.7 HU. In our study, both measurement methods showed good or excellent agreement for the liver and spleen in the pre-contrast and post-contrast CT images. Nonetheless, all volumetric measurements showed slightly lower values than the ROI-based measurements. This discrepancy may be attributed to the manual ROI placement, which selects relatively homogeneous areas, whereas volumetric measurements incorporate data from the entire organ volume. Thus, caution is warranted when considering a combined or alternating use of volumetric and ROI-based methods. Additionally, in automated segmentation-based volumetric analysis, caution is required in interpretation due to potential systematic errors of the algorithm from factors like image acquisition and anatomical variability. Implementing quality control and validation is essential to minimize these errors.

This study had some limitations. First, this study focused on a population of potential liver donor candidates, mostly in good health, which may limit the generalizability, especially considering the potential impact of concurrent conditions such as iron overload on CT attenuation values. However, this specific inclusion criterion was chosen to retrospectively assess the diagnostic value of organ segmentation-derived CT parameters for hepatic steatosis, using the well-established reference standard, MRS-PDFF, readily available for these subjects. Second, all included CT scans followed the same scan protocol from a vendor. Thus, further research involving varying scan settings and machines is needed to ensure the broad applicability of these findings. Third, this study adopted a cross-sectional design and did not explore the feasibility of longitudinal follow-up. Further investigation is needed to assess the utility of automated volumetric CT measurements in a population with repeated CT data to longitudinally monitor hepatic steatosis.

In conclusion, automated 3D segmentation of the liver and spleen in CT scans can provide volumetric CT attenuation-based parameters to detect and grade hepatic steatosis, applicable to pre-contrast and post-contrast images. This automated approach holds promise as an efficient alternative to manual ROI-based assessment, especially when dealing with large cohort populations.

### Supplementary Information

Below is the link to the electronic supplementary material.

Supplementary file1 (PDF 227 KB)

## Abbreviations

AUC: Area under the ROC curve
CI: Confidence interval
CT: Computed tomography
HU: Hounsfield unit
ICC: Intraclass correlation coefficient
MRS: Magnetic resonance spectroscopy
PDFF: Proton density fat fraction
ROC: Receiver operating characteristic
ROI: Region-of-interest

## Declarations

### Guarantor

The scientific guarantor of this publication is Ijin Joo.

### Statistics and biometry

No complex statistical methods were necessary for this paper.

### Methodology

• retrospective

• cross-sectional study

• performed at one institution
