# Supplementary material for: Utilizing fully-automated 3D organ segmentation for hepatic steatosis assessment with CT attenuation-based parameters
Source: Eur Radiol. 2024 Feb 23;34(9):6205–13. doi: 10.1007/s00330-024-10660-4 (PMC11364604; doi:10.1007/s00330-024-10660-4)
Supplement: Supplementary file 1 — Supplementary file1 (PDF 227 KB) [file 330_2024_10660_MOESM1_ESM.pdf]

**Utilizing Fully-Automated 3D Organ Segmentation for Hepatic Steatosis Assessment with CT Attenuation-Based Parameters**

**ELECTRONIC SUPPLEMENTARY MATERIAL**

## Supplementary Material 1. Image Acquisition

### Liver CT

Quadruple-phasic liver CT scans were performed using a Siemens Healthineers SOMATOM Force dual-energy CT scanner. The scans utilized a tube potential pair of 80/150 with a tin filter, with the A-tube set to 250 mAs and the B-tube to 125 mAs. The detector configuration was 192 × 0.6 mm, with a gantry rotation time of 0.5 s, a pitch of 0.6, and a 3 mm slice thickness (2 mm reconstruction intervals). All scans were reconstructed into a blended image with a 60% 80 kVp and 40% tin-filtered 150 kVp mix. Iobitridol (Xenetix 350, Guerbet) was intravenously injected at a dose of 520 mg/kg body weight using a power injector (Stellent D, Medrad) over 30 s at a rate of 2–5 mL/s, followed by a 20–30-mL saline flush. The arterial phase scan began automatically, 17 s after the abdominal aorta reached 80 HU at 150 kVp using the care bolus technique. Portal venous and delayed phase images were acquired 70 s and 180 s after contrast media administration, respectively. Portal venous phase scanning covered the dome of the liver to the pelvic inlet level, while pre-contrast, arterial, and delayed phase scans included the entire liver from the dome to the inferior tip.

### MRS-derived proton density fat fraction

All $^{1}$H-MRS examinations were performed using a clinical 3.0-T MR scanner, the Siemens Healthineers MAGNETOM Skyra, equipped with a 60-channel torso phased-array coil. The acquisition process involved obtaining sagittal, coronal, and axial sections covering the entire liver to position the spectroscopy acquisition voxel accurately. Then, a 3×3×3 cm signal voxel was placed in the dome area of segment VII or VIII of the liver, carefully avoiding large blood vessels, bile ducts, and liver edges. Fat fraction spectroscopy measurements were conducted with a modified stimulated-echo acquisition sequence at various echo times (12, 24, 36, 48, and 72 ms) within the single voxel. Each acquisition was completed in a 15-s breath hold. To calculate the proton density fat

fraction (PDFF), T2 correction was applied to both water and lipids, with separate T2 values calculated for each. Fat quantification was achieved through an exponential fit of data points acquired at different echoes, extrapolating fat and water integrals to an echo time of 0 ms. The resulting PDFF was automatically displayed as a percentage.

**Supplementary Figure 1**. Bland-Altman plots of volumetric versus region-of-interest-based measurement of CT attenuation values of the liver (**A:** pre-contrast CT, **C:** post-contrast CT) and spleen (**B**: pre-contrast CT, **D:** post-contrast CT).

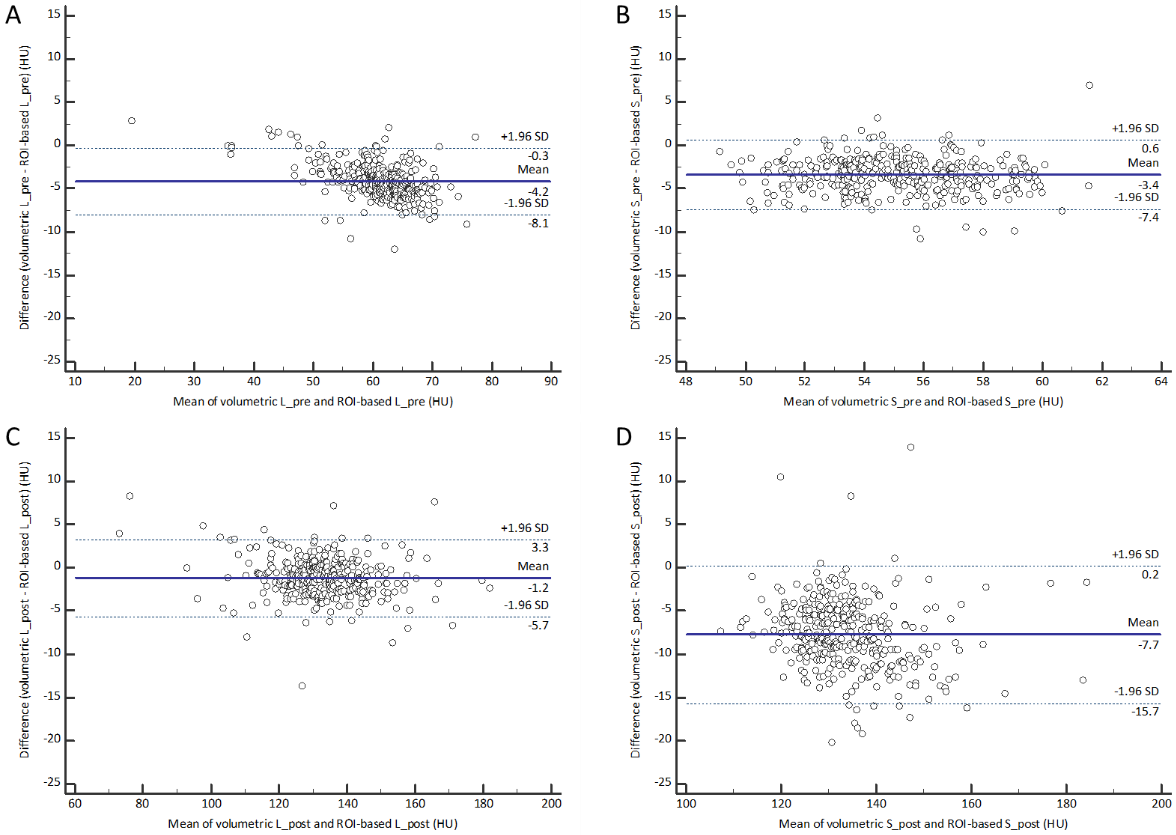

**Supplementary Table 1.** P values for comparing diagnostic performances of volumetric CT attenuation-based parameters

| Volumetric CT attenuation-based parameters | *p*-value | |
|---|---|---|
| | MRS-PDFF ≥5% | MRS-PDFF ≥10% |
| L_pre versus L-S_pre | 0.994 | 0.513 |
| L_post versus L-S_post | 0.001* | 0.144 |
| L_pre versus L_post | 0.015* | 0.052 |
| L-S_pre versus L-S_post | 0.898 | 0.150 |

Note. MRS-PDFF = magnetic resonance spectroscopy-proton density fat fraction; L_pre and L_post = mean liver HU on pre- and post-contrast CT images, respectively, L-S_pre and L-S_post = difference in mean HU between the liver and the spleen on pre- and post-contrast CT images, respectively. $^{*}p < 0.05$

Eur Radiol (2024) Yoo J, Joo I, Jeon SK, Park J, Yoon SH.
